# Ultrasonic seed treatment improved seed germination, growth, and yield of rice by modulating associated physio-biochemical mechanisms

**DOI:** 10.1016/j.ultsonch.2024.106821

**Published:** 2024-02-20

**Authors:** Suihua Huang, Umair Ashraf, Meiyang Duan, Yong Ren, Pipeng Xing, Zhuosheng Yan, Xiangru Tang

**Affiliations:** a State Key Laboratory for Conservation and Utilization of Subtropical Agricultural Bioresources, South China Agricultural University, Guangzhou 510642, China; b Institute of Quality Standard and Monitoring Technology for Agro-Products of Guangdong Academy of Agricultural Sciences, Guangzhou 510640, China; c Department of Botany, Division of Science and Technology, University of Education, Lahore 54770, Punjab, Pakistan; d Guangxi Key Laboratory of Agricultural Resources Chemistry and Biotechnology, Yulin Normal University, 537000, China; e Guangzhou Golden Rice Agricultural Science & Technology Co., Ltd., Guangzhou 510900, China

**Keywords:** Aromatic rice, Ultrasound, Hormones, Antioxidants, Physiology

## Abstract

Ultrasonic seed (US) treatment could alter seed germination mechanism, however, US induced alterations in morph-physiological attributes and yield of fragrant rice were rarely reported. In the present study, the seeds of three fragrant rice cultivars *viz.*, Xiangyaxiangzhan, Meixiangzhan 2, Ruanhuayou 6100 and one non-fragrant rice *viz.*, Wufengyou 615 were exposed to ultrasonic waves at 20–40 kHz for 1.5 min (T) whereas the seeds without exposure were taken as control (CK). Results showed that US treatment caused minor cracks on seed surface while improved seed germination rate (1.79 %-11.09 %) and 3-indoleacetic acid (IAA) (3.36 %-46.91 %). Furthermore, peroxidase (POD) activity and methionine sulfoxide reductase activity was increased by 29.15 %-74.13 % and 11.26 %-20.87 %, respectively; however, methionine sulfoxide reductase related protein repairing gene *MSRA4* was down-regulated by 17.93 % −41.04 % under T, compared to CK. Besides, US treatment also improved soluble protein in flag leaf (0.92 %-40.79 %), photosynthesis (3.37 %-16.46 %), biomass (5.17 %-31.87 %), as well as 2-acetyl-1-pyrroline content (4.77 %-15.48 %) in rice grains. In addition, multivariate analysis showed that the dry weight at the maturity stage were significantly related to the POD, glutathione reductase (GR) activity, IAA, and abscisic acid (ABA) content while germination rate was positively related to the GR activity, ABA content, and yield, but which were negatively related to the IAA and gibberellic acid content.

## Highlights

• US improved the rice germination rate in agriculture.
• US enhanced biomass and aroma by photosynthesis.
• The dry weight was strongly related to GR.

## 1 Introduction

Fragrant rice has its own repute owing to its unique scent and smooth texture [8], [2]. Among multiple compounds, the 2-acetyl-1-pyrroline (2-AP), one of the characteristic compounds in fragrant rice, which is responsible for the aroma in fragrant rice and can be substantially affected by internal and external plant factors [43], [27], [16], [3]. Generally, rice yield and quality characters are related to vegetative and reproductive growth that determined by nutrient uptake and use efficiencies [10]. For instance, nitrogen [14] or selenium and silicon [22] application improved the yield and lodging resistance in fragrant rice cultivars whereas foliar Zn [5] or Mn [20] application enhanced the grain 2AP contents by modulating its related regulatory mechanism.

Vigorous seeds are important regarding healthy seedlings, potentially leading to improvement in the final crop harvest [38], [42]. On the other hand, the germination process is often regulated by hormones such as gibberellin (GA) and abscisic acid (ABA), whereas water uptake slowed down at the post-germination stage, modulating seed metabolism and enzyme activities for radicle growth [6], [7]. However, the quality and viability of seeds could be inevitably harmed by temperature, moisture, microorganisms, and storage time [36], Zhao et al. [44], [17]. Internal physiological manifestations of seed aging, such as DNA damage [19], disruption of antioxidative system [32], hormones imbalance [18], and plasma membrane damage Wojtyla et al. [40], ultimately causes delay in seed germination.

Seed treatments are intentionally employed through biological, chemical, and physical approaches to improve seed vigor and emergence. Ultrasonic seed treatment is one of the physical approaches to break seed dormancy [36] and improve germination rate [33], or even strengthen the tolerance of rice plants under abiotic stress conditions [31]. Previous studies reported that the elastic mechanical waves generated during ultrasonic treatment caused changes in the structure and function of plant cells, plant metabolism, and enzymatic and physiological reactions to stimulate cell division [30], as well as improved cell viability [34], and accelerated plant growth and development [35]. In addition, ultrasonic seed treatment altered cell membrane permeability, accelerated seed swelling, and promoted starch hydrolysis as well, accordingly improving rice yield by increasing effective panicles and grains per panicle [4].

Previous studies have reported the positive effects of ultrasonic seed treatment on seed germination rate [12] and rice yield [31]; however, the effects of ultrasound in altering the ultra-structure of the seed coat surface, the physiology of germination, seedling, and biomass throughout the growth period of fragrant rice were rarely reported. The present study was therefore conducted to observe the effects of ultrasonic seed treatment on ultra-structures of the seed surface, biochemical mechanisms involved in germination process, plant metabolites, biomass, and yield of fragrant rice.

## 2 Materials and methods

### 2.1 Experimental set up

Seeds of three fragrant rice cultivars *viz.,* Xiangyaxiangzhan (Xiangsimiao 126 × Xiangyaruanzhan), and Meixiangzhan 2 (Lemont × Fengaozhan), the temperature-sensitive conventional rice types, and Ruanhuayou 6100 (G Ruanhua × R6), the temperature-sensitive hybrid rice type as well as one non-fragrant rice *viz.,* Wufengyou 615 (Wufeng A × Guanghui615), the temperature-sensitive hybrid rice type, were provided by the College of Agriculture, South China Agricultural University (SCAU) and Rice Research Institute Guangdong Academy of Agricultural Sciences, China. The seeds were pre-treated under ultrasonic waves by an Ultrasonic Crop Production Tunnel Processor for 1.5 min with 20–40 kHz mixed frequency (5ZCG-50, Guangzhou Golden Rice Agricultural Science & Technology Co., Ltd., Guangzhou, China), regarded as treatment (T). Seeds without ultrasonic treatment were taken as control (CK).

The treated seeds were then sterilized with 0.5 % NaClO solution for 5 min, rinsed by tap water, and placed on filter paper in glass dishes (diameter: 9 cm). The glass dishes were placed in an incubator at 28 °C in dark for 7 days and applied with enough water to keep the filter paper wet (total applied water was 20 ml). Well germinated seeds were sown into plastic boxes (35 cm × 25 cm) with substrate for seedling and placed in a growing chamber at 28 °C in light (3000 lx, 12 h) and dark (12 h) with 80 % relative humidity. Each group was in 6 dishes (100 seeds per dish) for germination and 6 dishes (100 seeds per dish) for seedlings.

Sterilized seeds for the pot experiment were sown on July 16th, 2018 in wet bed nurseries, and 4 seedlings per hill (5 hills per pot) were transplanted on July 30th, 2018 in a greenhouse at Experimental Farm, SCAU, Guangzhou, China (23°09′N, 113°22′E). The experimental soil was comprised of 22.41 g/kg organic matter, 1.01 g/kg total nitrogen, 0.82 g/kg total phosphorus, 23.56 g/kg total potassium, 86.68 mg/kg available nitrogen, 48.30 mg/kg available phosphorus, 68.12 mg/kg available potassium with 6.41 pH. The pots were applied with urea (181 mg/kg), calcium superphosphate (333 mg/kg), and potassium chloride (117 mg/kg) as 70 % basal dose at 3 days before transplanting, and the remaining 30 % was applied at tillering stage (August 29th, 2018). The total application amount of the fertilizer was 7.2 g per pot based on the recommended fertilizer dose on filed area basis (the plane area of the upper edge of the pot was 0.07 m^2^). The water layer was maintained at 2–3 cm above the soil until 4 days before harvest. The rice was harvested on November 2nd, 2018.

### 2.2 Plant sampling

Fresh seeds at 0 h, 24 h, and 72 h after soaking, top leaves at seedling stage, tillering, and booting stage, and flag leaves at heading stage, and maturity were sampled carefully, and stored at −80 °C for biochemical parameters. The above-ground plants were sampled at tillering, booting, heading and maturity stage, dried at 80 °C till constant weight for dry biomass. The grains at 15 days after full heading stage and maturity stage as well as milled rice were stored at −20 °C for 2AP estimation.

### 2.3 Endogenous hormones content

The contents of endogenous ABA, GA_3_, 3-indoleacetic acid (IAA), jasmonic acid (JA), and salicylic acid (SA) in seeds were determined by Suzhou BioNovoGene Biomedical Tech Co., LTD (Suzhou, China). Seeds without husk (100 mg) were extracted with 1 ml acetonitrile: water (1:1) solution with a small amount of sodium diethyl dithiocarbamate on ice for 4 h, and then, were centrifuged at 12000 rpm for 10 min at 4 °C. The supernatant was analyzed by ultra-performance liquid chromatography (AcQuity UPLC, Waters, USA).

### 2.4 Ultrastructure of seed pericarp

For scanning electron microscopy (SEM) observation, the dry seeds and the seeds at 72 h after soaking were kept in 2.5 % glutaraldehyde solution and vacuum extracted. The SEM images of the pre-treated samples were detected by Wuhan PorNetsBio Co., Ltd. (Wuhan, China) to observe the ultrastructure.

### 2.5 Germination rate and electric conductivity

The germination dynamics was recorded for six consecutive days from the start of seed germination. The germination rate was calculated according to the below formula:

Thegerminationrate(%)=thetotalnumberofgermintedseeds/thetotalnumberoftestedseeds×100%

The germination potentiality was calculated by the following formula:

$$The\;germination\;percentrage\;\left(\%\right)=the\;number\;of\;\;germinated\;seeds\;on\;the\;4^{th}\;day\;after\;germination/the\;total\;number\;of\;tested\;seeds\times 100\;\%\;\;$$

The germination index was calculated according to the below formula:

$The\;germination\;index=\sum_{n=0}^{t-1}Xn\left(t-n\right)/t;$*n*: the *n*^th^ day of germination minus 1; *t*: the total number of germination days; *Xn*: the number of germinated seeds on the *n*^th^ day.

The electric conductivity was determined by the method of Akdemir Evrendilek et al. [1]. About 25 g seeds were placed into plastic jars and filled with 250 ml distilled water at room temperature, measured by an electrical conductivity (DDS-303A, Leici, China).

The electric conductivity was calculated by the following formula:

Electric conductivity (μS/(cm·g)) = electric conductivity of (samples -water) / sample weight.

### 2.6 Real-time quantitative RT-PCR (qRT-PCR)

Fresh samples (two seeds) at 24 h and 72 h were grounded into powder, kept in an ice bath for 5 min after adding 1.5 ml TRE-Trizol and vortex mixed. For RNA extraction the HiPure Plant RNA Mini Kit (R4154-01, Magen, China) was used and the manufacturer’s protocols were followed. The quality of RNA was detected by an ultra-micro spectrophotometer (BioDrop Duo+, Biochrom Ltd, Germany). The cDNA was synthesized by HiScript II Q Select RT SuperMix for qPCR (+gDNA wiper) with 5 × HiScript® II Select qRT SuperMix II (R233-01, Vazyme, China) according to its protocol. The qRT-PCR analysis was conducted by qRT-PCR (CFX96, BIO-RAD, USA). Three replicates of each sample were set up with no cDNA template as a negative control, and *actin* was used as an internal reference gene. The PCR amplification rate was guaranteed to be between 95 % and 105 % and the relative gene expression was calculated using the method of ΔΔ Ct. The primer sequences of *OGG1*, *PIMT1*, *MSRA2.1*, and *MSRA4* were listed (Table S1).

### 2.7 DNA and protein repaired enzyme activities

Fresh samples (0.5 g) were ground into powder with liquid nitrogen, adding 9 ml sodium phosphate buffer (PBS, pH 7.4), the mixture was centrifuged at 3000 rpm for 20 min at 4 °C, and the supernatant was collected carefully. The activities of 8-oxoguanine DNA glycosylase (OGG1), protein L-isoaspartyl methyltransferase (PIMT1), and methionine sulfoxide reductase were determined by using plant tissue test kits according to protocols purchased from Shanghai Enzyme-linked Biotechnology Co., Ltd. (Shanghai, China).

### 2.8 Anti-oxidant and metabolic enzyme activities

Fresh seeds (0.5 g) were homogenized with 9 ml of 50 mM PBS (pH 7.8) in an ice bath and centrifuged at 8000 rpm for 15 min at 4 °C and the supernatant was obtained for measurement of anti-oxidant and metabolic enzyme activities. Peroxidase (POD) and catalase (CAT) activity were measured following the method of Huang et al. [15]. For POD, 0.05 ml of the supernatant mixed with 1 ml of PBS (pH 7.8), 0.95 ml guaiacol, and 1 ml of 0.3 % H_2_O_2_ and the absorbance was read at 470 nm. The POD activity was expressed as U/(g·min) FW. For CAT, the reaction mixture was contained 1.95 ml distilled water, 1 ml 0.3 % H_2_O_2_, and 0.05 ml supernatant, and then the absorbance was read at 240 nm. The CAT activity was expressed as U/(g·min) FW.

Fresh seed samples (0.5 g) were homogenized with 9 ml 50 mM PBS (pH 7.4) and centrifuged for 20 min at 3000 rpm at 4 °C, the supernatant was collected carefully. Methionine sulfoxide reductase (MSRA), 3-mercaptopyruvate sulfurtransferase (3-MST), glucose 6-phosphate dehydrogenase (G6PDH), glutathione reductase (GR) activities from the above supernatant were determined by using plant tissue test kits purchased from Shanghai Enzyme-linked Biotechnology Co., Ltd. (Shanghai, China) according to protocols. The MSRA activity, 3-MST activity, and GR activity were expressed as mU/g FW, and the G6PDH activity was expressed as U/g FW.

The α-Amylase activity was determined according to Li et al. [21] with some modifications. Fresh samples (1.0 g) were ground with 8 ml ultra-pure water and oscillated for 20 min at room temperature, and centrifuged at 3000 rpm for 10 min. The supernatant was then transferred to a 100 ml volumetric flask to obtain the original amylase solution. Solution was placed in water bath at 70 °C for 15 min, and 10 min at 40 °C with 1 ml 1 % starch solution, and then 5 min at 40 °C with subsequent addition of 2 ml 3,5-dinitrosalicylic acid (DNS) regent. After boiling for 10 min, the solution was cooled down, and 20 ml of ultra-pure water was added and the absorbance was read at 540 nm.

### 2.9 Soluble protein, proline and GSH content

Fresh leaf samples (0.5 g) were ground up with 9 ml of 50 mM PBS (pH 7.8) in an ice bath. The solution was centrifuged at 8000 rpm for 15 min at 4 °C and the supernatant was obtained for measurement. Soluble protein contents were estimated according to Huang et al. [15]. The 5 ml of Coomassie Brilliant Blue (G250) reagent was vortexed with 0.9 ml of ultra-pure water, and 0.1 ml of the supernatant and and the absorbance was read at 595 nm. The proline and GSH content were measured according to Rao et al. [31].

### 2.10 Photosynthesis and biomass

Photosynthesis, assimilation rate, stomatal conductance, internal CO_2_, and transpiration rate was determined with portable photosynthesis system (LI-6400, LI-COR, USA) during 9:00–11:00 a.m. on sunny day at seedling, tillering, and heading stage.

For dry matter weight, above ground plant parts were weighted after drying in oven. Phase dry matter weight was calculated by following the given formula: $\;{Phase\;dry\;matter\;weight}_{A-B}=the\;dry\;matter\;weight\;at\;\;stage\;A-\;the\;dry\;matter\;weight\;at\;\;stage\;\;B$;

Phase growth rate was calculated by following the given formula:

$${Phased\;growth\;rate\;}_{A-B}={phase\;dry\;matter\;weight}_{A-B}/days\;between\;two\;sampled\;date$$

Tillering to booting stage: 18 d (days), booting to heading stage: 10 d, Heading stage to maturity: 28 d.

### 2.11 Yield, grains quality and 2AP content

The grains were harvested at maturity and sun-dried to 13–14 % of moisture content. The yield was obtained after estimating the number of effective panicles, grains per panicle, filled grain percentage, and 1000 grain weight per pot. The effective panicles were counted from each pot; grains were separated manually from each panicle and total and unfilled grains were counted. The 1000 grains were weighed for estimation of 1000 grain weight whereas the filled grain percentage was calculated by the following formula:

Filled grain percentage (%) = (total grain number-unfilled grain number)/total grain number × 100 %.

Grains from each group were sun dried and stored at room temperature for three months and then used to determine the grain quality. About 60 g grains were hulled into brown rice with a rice huller (OTAKE, Japan), and then milled with a rice miller (SDJ-100, China), and the fine rice was weighed and the milled rice percentage was calculated after removing the rice bran. The SC-E Rice Appearance Quality Tester (Wanshen, China) was used to assess the rice chalkiness. A near-infrared grain quality analyzer (Infratec TM1241, FOSS, Denmark) was used to evaluate protein, amylose and straight-chain starch.

About 5 g of grains at 15 d after full heading and maturity stage were milled and ground to estimate 2-AP contents. The 2-AP contents were estimated by synchronization distillation and extraction method (SDE) using Gas Chromatography Mass Spectrometer (GC/MS) system following the method of Mo et al. [27].

### 2.12 Statistical analyses

The experiments were arranged in a completely randomized design (CRD) in triplicate. Analysis of variance (ANOVA) was performed in Statistix version 8 (Analystical and Takkahassee, Florida, USA), using the least significant difference (LSD) test at level *P* < 0.05. Results were expressed as mean ± standard error. The detected data were imported into MetaboAnalyst software (https://www/metaboanalyst.ca; [28] for *meta*-analysis, including the correlation of heatmap, Principal Component Analysis (PCA), Partial Least Squares-Discriminant Analysis (PLS-DA), significant variance parameters in T-test at (LSD) level *P* < 0.01, Mean Decrease Accuracy and Hierarchical Clustering Dendrogram. GraphPad Prism 8 (GraphPad Software, USA) was used for graphical representation.

## 3 Results

### 3.1 SEM observation of seeds

**Fig. 1:**
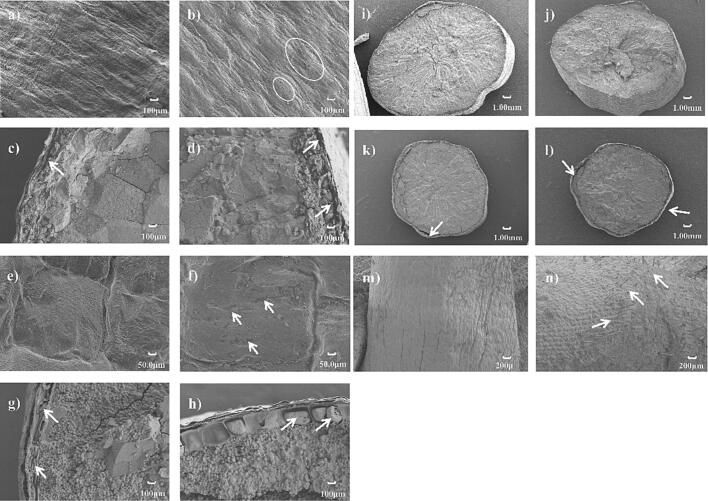
The pericarp ultrastructure (100 μm) of dry seed under a) CK, b) T treatment, cross section (100 μm) under c) CK, d) T treatment, the pericarp ultrastructure (50 μm) of rice seed at 72 h after seeds soaking under e) CK, f) T treatment, cross section (100 μm) under g) CK, h) T treatment. The ultrastructure of whole cross section (1.00 mm) of dry seed under i) CK, j) T treatment, the whole cross section (1.00 mm) of seeds at 72 h after seeds soaking under k) CK, l) T treatment, ultra ultrastructure of radicle (200 μm) at 72 h after seeds soaking under m) CK and n) T treatment.

Clearly visible reticulated ridges with reticulate ornamentation were observed on the ultrastructure of pericarp of the dry seed. Compared with CK, obvious cracks were noticed in the white circles (Fig. 1a and b). The pericarp, seed coat, nucellus, aleurone layer, and endosperm were obviously visible, respectively, and the white arrow pointing out that the gap between nucellus and aleurone layer under T was clearer than CK (Fig. 1c and d). At 72 h, the ultrastructure of pericarp was smoother and flatter than at 0 h than CK whereas a few fine traces were noticed (pointed out by the white arrows) under T (Fig. 1e and f). Furthermore, more swelling on aleurone layer with pores were observed under T than CK (Fig. 1g and h). Similar images from the cross section were noticed under CK and T at 0 and 72 h (Fig. 1i-l), however, more seed hairs on radicle at 72 h broke through under T than under CK (Fig. 1m and n).

### 3.2 Endogenous hormones content in seeds

Significant differences between treatments were detected in the endogenous hormones content (Fig. S1). There were similar trends in the tested cultivars that endogenous ABA and GA_3_ levels were decreased under T compared to CK. On the contrary, endogenous IAA levels were found higher in T than CK (Fig. S1a-c) during the seed germination period. Endogenous SA content was increased by 1.76 %-55.83 % in T than in CK at 0 and 24 h, but there was a significant decline at 72 h for all rice cultivars (Fig. S1d). Moreover, the GA_3_/ABA and IAA/ABA was remained 1.63 %-68.35 % and 7.26 %-120.46 % higher in T than CK during the germination period for all rice cultivars, respectively (Fig. S1f and g).

### 3.3 DNA and protein repairing gene expression and activity of seeds

**Fig. 2:**
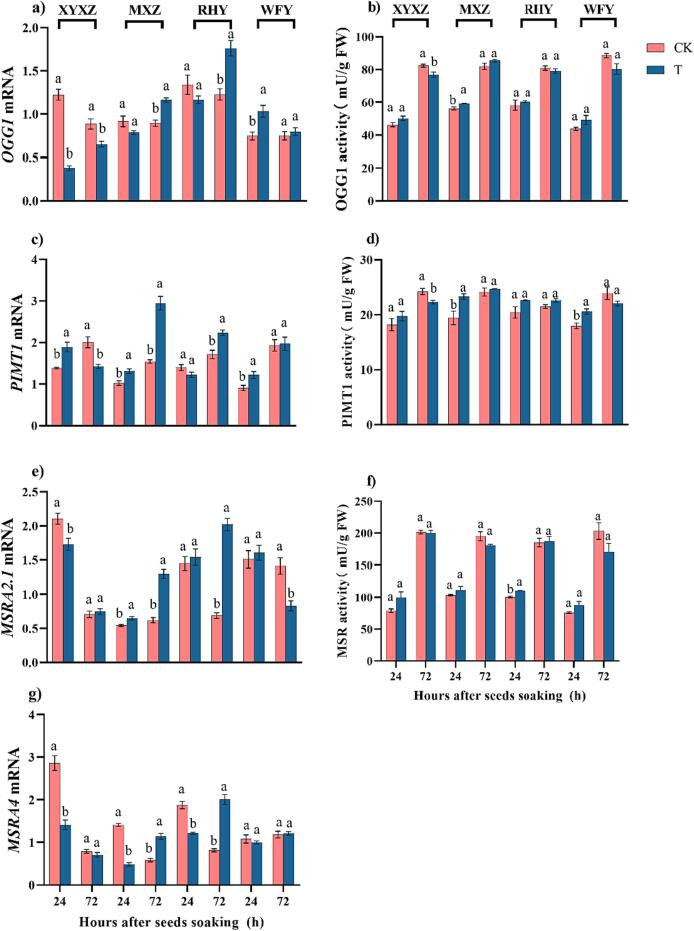
Effects of ultrasonic seed treatment on DNA and protein repairing gene expression of a) *OGG1*, c) *PIMT1*, e) *MSRA2.1*, g) *MSRA4*, the activities of b) OGG1, d) PIMT1, and f) MSR in seeds of four rice cultivars. Each column represented the mean ± standard error (n = 3). Bars sharing a common letter did not differ significantly at *p* < 0.05. OGG1: 8-oxoguanine DNA glycosylase, PIMT1: protein L-isoaspartyl methyltransferase, MSR: methionine sulfoxide reductase. CK: control treatment, T: ultrasonic seed treatment. XYXZ: Xiangyaxiangzhan, MXZ: Meixiangzhan 2, RHY: Ruanhuayou 6100, WFY: Wufengyou 615.

Variations in DNA and protein-repairing gene transcription levels and activities at 24 h and 72 h were detected for all rice cultivars (Fig. 2). Compared with CK, the expression of *OGG1* was down-regulated by T for Xiangyangzhan, Meixiangzhan 2, and Ruanhuayou 6100 with a range of 13.00 %-69.21 %, nevertheless, the activity of OGG1 was increased at 24 h but the opposite trend was noticed at 72 h (Fig. 2a and b). Compared to CK, the expression of *PIMT1* was substantially higher under T at 24 h and 72 h for Meixiangzhan 2 while the activity of PIMT1 showed a significant increase at 24 h (Fig. 2c and d). Compared with CK, a notable reduction in *MSRA2.1 and MSRA4* was noticed at 24 h for Xiangyaxiangzhan, however, a substantial increase was noticed at 72 h for Meixiangzhan 2 and Ruanhuayou 6100 was noticed. Moreover, no significant difference was found between CK and T of MSR activity for all rice cultivars (Fig. 2e-g).

### 3.4 Anti-oxidant enzymes activity of seeds

Ultrasonic seed treatment notably affected the activity of anti-oxidants in all rice cultivars (Fig. S2). The POD activity was significantly improved by T compared with CK within a range of 29.15 %-74.13 % across all rice cultivars whereas the MSRA activity also showed a similar trend (Fig. S2a and c). No significant difference regarding CAT activity was noticed between CK and T except for Wufengyou 615 at 72 h while a similar trend was found for α-amylase activity (Fig. S2b and e). Moreover, the G6PDH activity was remarkably decreased under T in Meixiangzhan 2 at 72 h, as compared with CK, however, no significant difference was noted between the two treatments for other rice cultivars (Fig. S2f). The 3-MST activity was improved by 2.32 %-28.47 % under T for all rice cultivars while the GR activity was increased by 8.31 %-15.59 % at 72 h under T compared with CK (Fig. S2d and g).

### 3.5 Germination rate and electric conductivity in seeds

The pictorial view of germinated seeds at 72 h and 120 h showed that T improved the seed germination and growth (Fig. S3a and b) with an increment by 1.35 %-4.24 % under T compared with CK (Fig. S3d). Compared to CK, the germination potentiality was substantially improved under T, which was increased by 18.02 %, 27.33 %, and 30.00 % for Xiangyaxiangzhan, Meixiangzhan 2, and Wufengyou 615, respectively (Fig. S3e). In addition, the T significantly reduced the relative conductivity for Xiangyaxiangzhan, Meixiangzhan 2, and Ruahuayou 6100 at 72 h than CK while increased in Wufengyou 615 (Fig. S3c).

### 3.6 Soluble protein, proline, GSH contents and amylase activity

Ultrasonic seed treatment remarkably improved the soluble protein, proline, GSH contents and amylase activity in the flag leaf (Fig. S4). Ultrasonic treatment led to an increase of 0.92 %-40.79 % in soluble protein, 0.18 %–33.28 % in soluble sugar, and 3.26 %-38.03 % in proline, respectively, than CK for all rice cultivars during the vegetative and reproductive growth period (Fig. S4a, b and d). The GSH content was increased by 0.45 %-35.12 % in T than CK for all rice cultivars during the vegetative and reproductive growth period (Fig. S4c). Moreover, amylase activity was increased by T for all rice cultivars at tillering, booting, heading, and maturity stage. For instance, the T improved 7.19 % for Xiangyaxiangzhan at tillering stage, 10.00 % for Ruanhuayou 6100 at booting stage, 3.68 % for Meixiangzhan 2 at heading stage, and 3.21 % for Wufengyou 615 at maturity, respectively (Fig. S4e).

### 3.7 Photosynthesis and plant biomass

Photosynthesis and plant biomass were significantly affected by ultrasonic seed treatment (Fig. S5). Substantial increments (3.37 %-16.46 %) in net photosynthesis were detected under T relative to CK at seedling, tillering, and heading stage, except for Xiangyaxiangzhan at the heading stage (Fig. S5a). The stomatal conductance at tillering stage was higher than other stages for all cultivars which was also increased by T compared with CK (Fig. S5b). Similar trends for internal CO_2_ and transpiration rate was observed for four rice cultivars at seedling, tillering, and heading stage (Fig. S5c and d).

Furthermore, during vegetative and reproductive growth period, dry biomass was significantly enhanced by T compared with CK within a range of 8.25 %-26.72 % from tillering to maturity stage for Xiangyaxiangzhan and Meixiangzhan 2 (Fig. S5e). Relative to CK, ultrasonic treatment notably increased the dry biomass from heading to the maturity stage and similar results were shown in phase growth rate (Fig. S5f and g).

### 3.8 Grain yield and quality traits

Ultrasonic seed treatment improved the yield and its related traits (Table 1). For Xiangyaxiangzhan, effective panicle number per pot, filled grain percentage, and grain yield were significantly improved but grains per panicle were notably reduced by T, compared with CK. For Meixiangzhan 2, similar results were shown regarding effective panicle number per pot, grains per panicle and grain yield in T, however, no significant difference between CK and T was noticed in Ruanhuayou 6100 regarding for yield and its related traits. Regarding Wufengyou 615, grains per panicle, filled grain percentage, and yield were remarkably increased by T than CK.

**Table 1:** Effects of ultrasonic seed treatment of yield and its related traits.

Cultivars	Treatments	Effective panicle number per pot	grains per panicle	Filled grain percentage (%)	1000 grain weight (g)	Yield (g/pot)
XYXZ	CK	17.05 ± 0.81b	116.26 ± 0.32a	94.72 ± 0.18b	18.81 ± 0.83a	35.23 ± 1.52b
	T	22.31 ± 0.79a	106.55 ± 0.23b	97.06 ± 0.16a	19.31 ± 0.04a	44.55 ± 1.55a
MXZ	CK	19.89 ± 0.50b	103.58 ± 0.21a	87.73 ± 1.19a	18.66 ± 0.03a	33.74 ± 1.28a
	T	22.74 ± 0.72a	99.53 ± 0.31b	90.96 ± 0.61a	18.68 ± 0.05a	38.46 ± 1.31a
RHY	CK	18.00 ± 0.58a	136.10 ± 1.22a	90.46 ± 1.44a	19.16 ± 0.16a	42.48 ± 1.79a
	T	17.00 ± 1.15a	136.34 ± 0.18a	92.72 ± 0.30a	19.24 ± 0.08a	41.35 ± 2.89a
WFY	CK	15.00 ± 0.58a	137.68 ± 1.76b	94.53 ± 0.17b	19.04 ± 0.26a	37.16 ± 1.45b
	T	16.33 ± 0.88a	150.21 ± 0.37a	97.59 ± 0.23a	18.92 ± 0.02a	45.29 ± 2.38a

Each column represented the mean ± standard error (n = 3). Bars sharing a common letter did not differ significantly at *p* < 0.05. CK: control treatment, T: ultrasonic seed treatment. XYXZ: Xiangyaxiangzhan, MXZ: Meixiangzhan 2, RHY: Ruanhuayou 6100, WFY: Wufengyou 615.

No significant difference in brown rice rate, milled rice rate, or protein contents was noticed under CK and T for all rice cultivars (Fig. S6a, b, and d). Notably, head rice rate and amylose contents of Meixiangzhan 2 were affected by T, compared with CK (Fig. S6c and e). Compared with CK, the ultrasonic seed treatment remarkably decreased alkali spreading value and chalkiness for Xiangyaxiangzhan (Fig. S6f and g). Additionally, the 2-AP content in milled rice was the lowest among three stages, however, ultrasonic seed treatment significantly increased the 2-AP content for Xiangyaxiangzhan and Meixiangzhan 2. Moreover, for Xiangyaxiangzhan, the grains was increased by 4.77 % and 15.48 % at 15 d after full heading stage and maturity stage whereas the milled rice was increased by 17.88 % under T than CK. For Mexianxiang 2, the the grains was increased by 17.59 % and 7.46 % at 15 d after full heading and maturity stage whereas the milled rice was increased by 20.86 % under T than CK (Fig. S6h).

### 3.9 Multivariate analysis

**Fig. 3:**
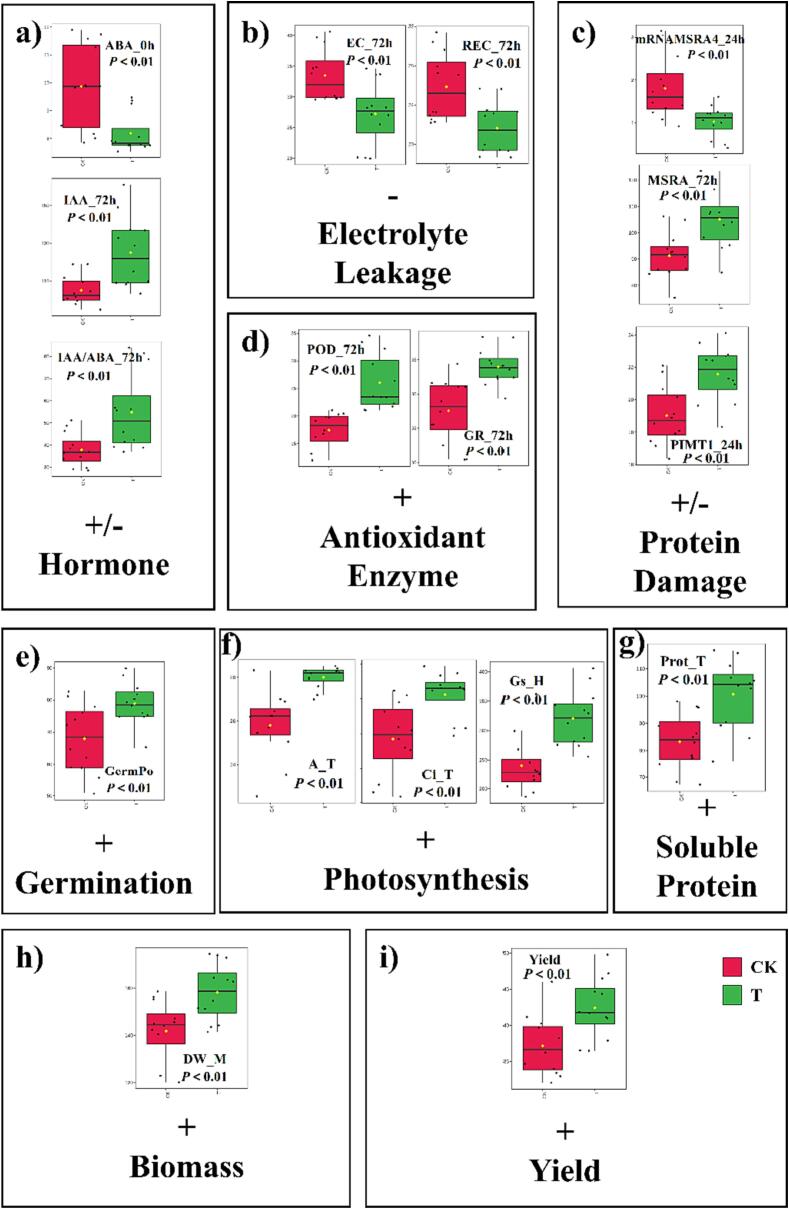
Effects of ultrasonic seed treatment on a) hormone contents, b) electrolyte leakage, c) protein damage repairing in seeds, d) antioxidant enzyme activities, e) germination, f) photosynthesis, g) soluble protein content in flag leaf, h) biomass and i) yield with detailed parameters changes. Each box and whisker plots represented the mean ± standard error (n = 12). *p* < 0.01 referred to significant variances between paired group in *t*-test (CK: control treatment and T: ultrasonic seed treatment). +: increased, -: decreased, +/-: changing by the periods. ABA_0h: abscisic acid content in seeds at 0 h after seeds soaking, IAA_72h: 3-indoleacetic acid content in seeds at 72 h after seeds soaking, IAA/ABA_72h: the ratio of 3-indoleacetic acid and abscisic acid content in seeds at 72 h after seeds soaking, EC_72h: electric conductivity in seeds at 72 h after seeds soaking, REC_72h: relative conductivity in seeds at 72 h after seeds soaking, mRNAMSRA4_24h: relative gene expression of *MSRA4* in seeds at 24 h after seeds soaking, MSRA_72h: methionine sulfoxide reductase activity in seeds at 72 h after seeds soaking, PIMT1_24h: protein L-isoaspartyl methyltransferase activity in seeds at 24 h after seeds soaking, POD_72h: peroxidase activity in seeds at 72 h after seeds soaking, GR_72h: glutathione reductase activity in seeds at 72 h after seeds soaking, GermPo: germination potentiality, Prot_T: soluble protein content in flag leaf at tillering stage, A_T: assimilation rate in flag leaf at tillering stage, Gs_H: stomatal conductance in flag leaf at heading stage, Ci_T: internal CO_2_ in flag leaf at tillering stage, DW_M: dry matter weight at maturity.

Generally, significant differences between CK and T were shown to provide evidence regarding T that led to an increase in yield for all rice cultivars (Fig. 3). The IAA content (hormone), POD, GR (antioxidant enzyme), MSRA, and PIMT1 (protein damage repairing enzyme) activity in seeds were significantly increased by T, leading to reductions of electrolyte leakage and increased germination (Fig. 3a-e). For vegetative and reproductive growth, the photosynthetic efficiency, soluble protein, and biomass were significantly enhanced by T, resulting in yield improvement (Fig. 3f-i).

**Fig. 4:**
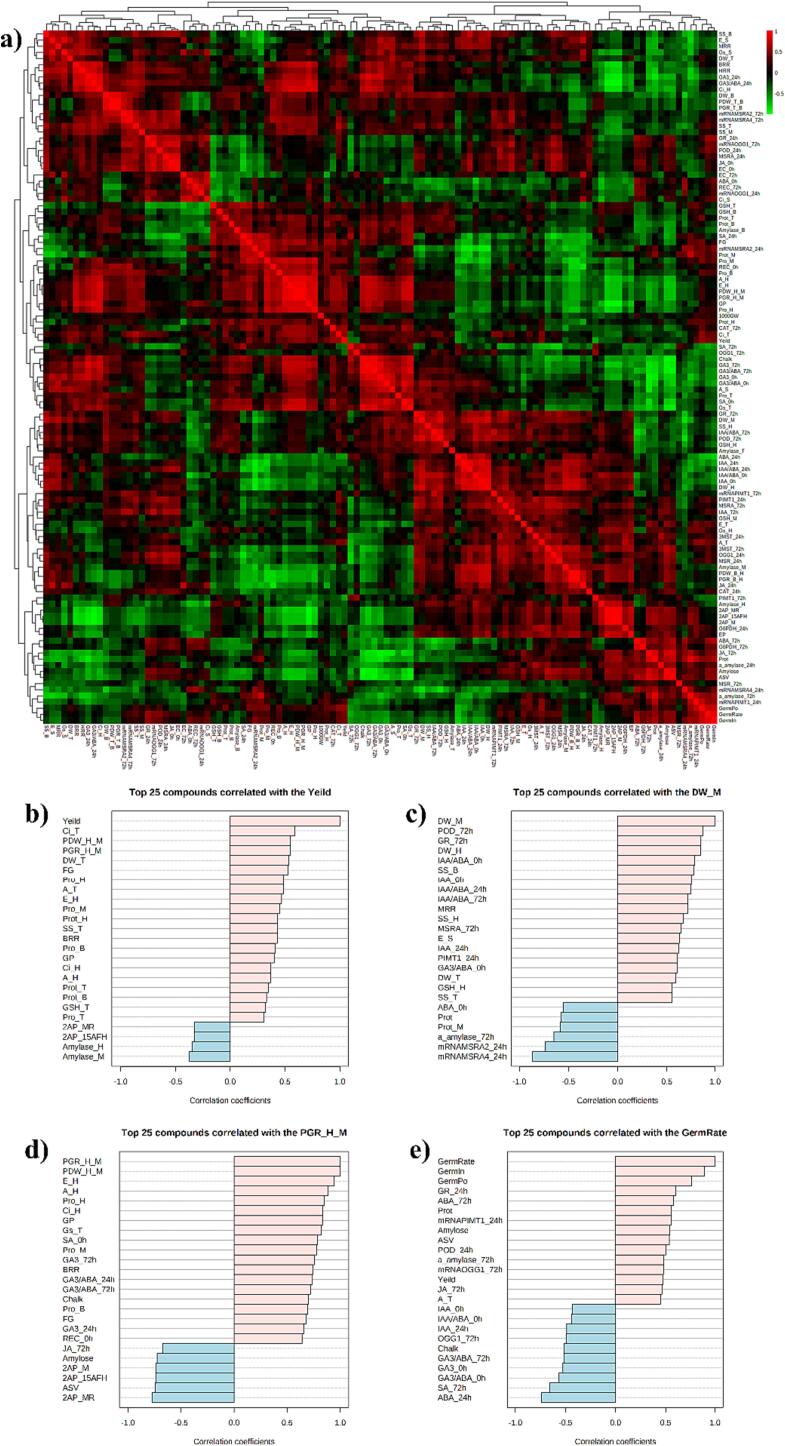
A) The heatmap in Pearson correlation analysis for investigated parameters. Top 25 compounds correlated with b) yield, c) DW_M, d) PGR_H_M and GermRate. DW_M: dry weight at maturity, PGR_H_M: phased growth rate from heading stage to maturity, GermRate: germination rate, GermPo: germination potentiality, GermIn: germination index, A_S: assimilation rate in flag leaf at seedling stage, A_T: assimilation rate in flag leaf at tillering stage, A_H: assimilation rate in flag leaf at heading stage, Ci_S: internal CO_2_ in flag leaf at seedling stage, Ci_T: internal CO_2_ in flag leaf at tillering stage, Ci_H: internal CO_2_ in flag leaf at heading stage, Gs_S: stomatal conductance in flag leaf at seedling stage, Gs_T: stomatal conductance in flag leaf at tillering stage, Gs_H: stomatal conductance in flag leaf at heading stage, E_S: transportation rate in flag leaf at seedling stage, E_T: transportation rate in flag leaf at tillering stage, E_H: transportation rate in flag leaf at heading stage, EC_0h: electric conductivity in seeds at 0 h after seeds soaking, EC_72h: electric conductivity in seeds at 72 h after seeds soaking, REC_0h: relative conductivity in seeds at 0 h after seeds soaking, REC_72h: relative conductivity in seeds at 72 h after seeds soaking, DW_T: dry matter weight at tillering stage, DW_B: dry matter weight at booting stage, DW_H: dry matter weight at heading stage, DW_M: dry matter weight at maturity, PDW_T_B: phased dry matter weight from tillering to booting stage, PDW_B_H: phased dry matter weight from booting to heading stage, PDW_H_M: phased dry matter weight from heading stage to maturity, PGR_T_B: phased growth rate from tillering from booting stage, PGR_B_H: phased growth rate from heading stage from maturity, BRR: brown rice rate, MRR: milled rice rate, HRR: head rice rate, EP: effective panicle number per pot, GP: grains per panicle, FG: filled grain percentage, 1000GW: 1000 grain weight, Prot: protein in grains, ASV: alkali spreading value, Chalk: chalkiness, Prot_T: soluble protein content in flag leaf at tillering stage, Prot_B: soluble protein content in flag leaf at booting stage, Prot_H: soluble protein content in flag leaf at heading stage, Prot_M: soluble protein content in flag leaf at maturity, Pro_T: proline content in flag leaf at tillering stage, Pro_B: proline content in flag leaf at booting stage, Pro_H: proline content in flag leaf at heading stage, Pro_M: proline content in flag leaf at maturity, SS_T: soluble sugar in flag leaf at tillering stage, SS_B: soluble sugar in flag leaf at booting stage, SS_H: soluble sugar in flag leaf at heading stage, SS_M: soluble sugar in flag leaf at maturity, Amylase_T: amylase activity in flag leaf at tillering stage, Amylase_B: amylase activity in flag leaf at booting stage, Amylase_H: amylase activity in flag leaf at heading stage, Amylase_M: amylase activity in flag leaf at maturity, 2AP_15AFH: 2AP content in grains at 15 d after full heading stage, 2AP_M: 2AP content in grains at maturity, 2AP_MR: 2AP content in milled rice.

The Pearson correlation amongst the investigated parameters in CK and T treatments for all rice cultivars was performed (Fig. 4a). To further concise the investigated parameters, the top 25 parameters that related to germination rate and grain yield were selected. It was found that the photosynthetic efficiency, phrased growth rate, filled grains, proline, and soluble protein were positively associated with yield (Fig. 4b). The POD, GR activity, IAA, and ABA content were significantly related to the dry weight at the maturity stage (Fig. 4c) while phase growth rate, photosynthesis efficiency, and GA_3_ content were strongly connected with the phase growth rate from heading to maturity stage (Fig. 4d). Moreover, germination rate was positively related to germination potentiality, germination index, GR activity, ABA content, genes encoding repaired protein damage, and yield; however, the IAA and GA_3_ content were negatively related to the germination rate (Fig. 4e).

In order to study the core parameters, the PCA and PLS-DA analysis were conducted. The PCA analysis revealed that the PC1, PC2, PC3, PC4, and PC5 respectively accounted for 97.5 %, 1.5 %, 0.3 %, 0.2 %, and 0.1 %, respectively while the PLS-DA showed that the component 1, component 2, component 3, component 4, and component 5 were 97.5 %, 1.4 %, 0.4 %, 0.3 %, and 0.1 %, respectively. The clusters of four cultivars in CK and T were performed separately (Fig. S7a-c and k). Regarding the components, the key parameters like the stomatal conductance, SA, GSH, 2-AP remained high levels under T compared with CK. Moreover, the coefficients of stomatal conductance, SA, 2-AP content exceeded 40. The parameters ranked by their contributions to mean decreased accuracy showed that POD activity in seeds, photosynthesis efficiency needs further investigation (Fig. S7d-j).

## 4 Discussion

Previous study indicated that the cavitation caused by ultrasonic treatment potentially had mechanical pressure on the seed, leading to some pores on the surface of the seed [24]. In the present study, a few cracks were observed on the pericarp with a reducing relative electric conductivity at 72 h for the fragrant rice (Fig. S3), which corroborates with Ding et al. [12] who reported that the ultrasonic seed treatment appropriately damaged the seed surface or softened the seed coat by the owing to cavitation collapse, producing more opportunities for water and oxygen uptake. Accelerated germination possibly corresponds to the swelling of aleurone layers and developing radicles under ultrasonic seed treatment (Fig. 1), assuming that the energy transmitted into the seeds and the water possibly stimulated the seed germination process; however, there were some physiological modifications but no significant morphological changes were noted on the seed surface of soybeans under the ultrasonic frequency at 25 kHz for 25 min i. e., imperceptible pores in nano-dimensions probably formed by the energy and cavitation were not excluded [23].

Furthermore, hormones play an important role to break seed dormancy and stimulation of seed germination process [33]. The swelling aleurone grains normally caused by the programmed cell death induced by the antagonism and synergy of ABA and GAs (Wojtyla *et al.* 2016b) which were in line with our present results (Fig. S1f). During germination, the endogenous hormones may interactively accelerate the seed germination process. Our results reflected that T could break the dormancy and stimulate the seed germination process. Besides, induced GA_3_ was released to the aleurone cells by embryo to biosynthesize the α-amylase and hydrolyze the starch, ending up with a reduction of GA_3_ [39]. Similarly, our study showed a decline in GA_3_ and an increase in α-amylase under ultrasonic seed treatment (Fig. S2e). Previously, Zhao et al. [45] reported that the IAA priming enhanced the germination and seedling growth by regulating the endogenous hormones which was in agreement with our results (Fig. S1). Correspondingly, the germination potentiality was higher in ultrasonic seed treatment than in CK (Fig. 1e) which corroborates with the study of Sharififar et al. [33] who reported that an appropriate ultrasonic exposure positively affected the seed germination. Therefore, it can be assumed that T could regulate the balance between IAA, ABA, and GA_3_ where the level of GA_3_ was also associated with the biosynthesis of α-amylase.

High vigor of seeds potentially represents the stable and rapid emergence of seeds under field conditions [38]. Activation of DNA repairing mechanism is important to improve the seed vigor during the imbibition whereas the *OGG1* (up-regulated under oxidase stress) removes 7,8-dihydro-8-oxoguanine (8-oxo-dG) in the base excision repair pathway [26]. The *PIMT* was found to catalyze the conversion of L-isoaspartate (L-isoAsp) to normal L-aspartate, inhibiting protein misfolding caused by L-isoAsp. Up-regulation in *PIMT1* was observed in this study (Fig. 2c) which is in accordance with Wei et al. [37] who found that overexpression of *OsPIMT1* in transgenic rice seeds reduced isoAsp in embryos and increased embryo viability [37]. Moreover, the *MSRA4* were significantly down-regulated at 24 h but up-regulated at 72 h under ultrasonic seed treatment with an improvement in MSRA activity (Fig. 2g and S2c). The *MSRAs* might have stimulated by endogenous hormones [11] whilst higher antioxidants activities could improve the tolerance of oxidant damage [13].

Ultrasonic waves contain energy which may transfer into the seeds. The present study revealed that higher antioxidant enzyme activities, such as POD, GR, and 3-MST, were in ultrasonic seed treatment while the highest electrolyte leakage was observed at 0 h and the lowest at 72 h (Figs. S2 and S3). It was hypothesized that the ultrasonic treatment damaged the plasma membrane, however repaired quickly owing to the improvement in antioxidant enzyme activities. The mechanical oscillation and cavitation effects of ultrasonic waves enhanced the activities of enzymes to catalyze substrate [41] whilst the released energy changed the conception of the enzyme molecule to a reasonable degree to improve the antioxidant enzyme activity [9]. Moreover, the metabolic enzyme activities of GR and α-amylase were enhanced by ultrasonic seed treatment (Fig. S2e and g). On the other hand, instantaneous amount of electrolyte leakage stimulated antioxidant enzymatic system by enhancing the activities of POD and MSRA (Fig. S2a and c). Our results regarding DNA, protein repair, and antioxidant defense suggested that the exposure to ultrasonic waves moderately damaged the plasma membrane of seeds, which in turn triggered the plant defense mechanisms and led to enhanced growth and yield development.

Previously, Zhong et al. [46] revealed that the rice reduced its nitrogen allocation to maintain photosynthesis system with a higher level of soluble protein under stress, which was positively correlated with photosynthetic nitrogen use efficiency. Herein, the remarkable enhanced soluble protein content in leaves at tillering stage (Fig. S4a) might contributed to the traits of photosynthesis, i.e., assimilation rate, stomatal conductance, and internal CO_2_ (Fig. S5a-c) under ultrasonic seed treatment. Furthermore, the dry biomass at each growing stage was higher in ultrasonic seed treatment than in CK (Fig. S5e-g), leading to improved grain yield (Table 1). Moreover, the soluble protein content, photosynthetic efficiency, and phase growth rate was positively correlated with the grain yield (Fig. 4). These findings were in line with the earlier study of Perveen et al. [29] who found an improvement in the photosynthetic capacity was essentially contributed to the enhanced grain yield in rice. It was reported that higher rate of photosynthesis also contributed to the enhanced 2-AP levels in fragrant rice [25] that corroborates with our findings (Fig. S6h and S7f-h). In this study, the performance of seeds, seedlings, and biomass accumulation under ultrasonic seed treatment were better than CK owing to improved efficiency of antioxidants and photosynthetic system however, the relationship between SA and 2-AP for rice quality (Fig. S7f-g) needs further investigations due to the positive production of primary metabolites.

**Fig. 5:**
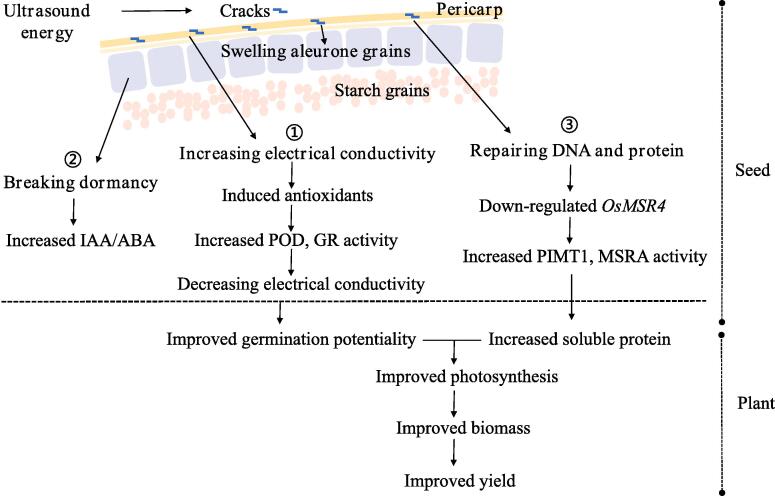
Hypothetical mechanism of ultrasonic seed treatment improving germination and growth in fragrant rice. ABA: abscisic acid, IAA: 3-indoleacetic acid, POD: peroxidase, GR: glutathione reductase, PIMT1: protein L-isoaspartyl methyltransferase, MSRA_72: methionine sulfoxide reductase.

In addition, a hypothetical model was performed to explain how ultrasonic seed treatment improved the germination, growth, and yield of rice at a physiological level (Fig. 5). In brief, the ultrasound energy generated a few cracks on the surface of the pericarp, enhancing the water absorption which accelerated the process of programmed cell death. Furthermore, a germination-promotive effect of IAA and ABA was synergistically triggered to break the seed dormancy after seed imbibition. Besides, the down-regulation of *OsMSR4* increasing the activities of PIMT1 and MSRA, participated in repairing DNA and protein in seeds, which improved the germination potentiality. In addition, as a result of improved germination and seedling growth, the increased net photosynthetic rate contributed to better biomass accumulation and grain yield.

## 5 Conclusion

Ultrasonic seed treatment in 20–40 kHz mixed frequency for 1.5 min had a positive impact on germination and growth of fragrant rice owing to modulation in seed morphological and physiological attributes. Ultrasonic seed treatment also enhanced the activities of POD and GR whereas down-regulation in *MSR4* was related to higher MSRA activity which repaired the protein in seeds. Overall, improved germination potentiality and increased soluble protein in leaves jointly improved the photosynthetic efficiency which contributed to the improved biomass and grain yield of rice.

## Funding sources

This work was supported by the National Natural Science Foundation of China 31971843 and the Natural Science Foundation of Guangdong Province 8151064201000017.

## CRediT authorship contribution statement

**Suihua Huang:** Writing – original draft, Visualization, Investigation, Formal analysis, Conceptualization. **Umair Ashraf:** Writing – original draft, Investigation, Formal analysis. **Meiyang Duan:** Writing – review & editing, Resources, Formal analysis. **Yong Ren:** Validation, Investigation, Formal analysis. **Pipeng Xing:** Investigation. **Zhuosheng Yan:** Resources. **Xiangru Tang:** Writing – review & editing, Supervision, Funding acquisition, Conceptualization.

## Declaration of competing interest

The authors declare that they have no known competing financial interests or personal relationships that could have appeared to influence the work reported in this paper.
