# Supplementary material for: Ultrasonic seed treatment improved seed germination, growth, and yield of rice by modulating associated physio-biochemical mechanisms
Source: Ultrason Sonochem. 2024 Feb 20;104:106821. doi: 10.1016/j.ultsonch.2024.106821 (PMC10901143; doi:10.1016/j.ultsonch.2024.106821)
Supplement: Supplementary data 1 [file mmc1.docx]

**Ultrasonic seed treatment improved seed germination, growth, and yield of rice by modulating associated physio-biochemical mechanisms**

Suihua Huang^1,2^, Umair Ashraf^3^, Meiyang Duan^1^, Yong Ren^4^, Pipeng Xing^1^, Zhuosheng Yan^5^, Xiangru Tang^1#^

^1^ *State Key Laboratory for Conservation and Utilization of Subtropical Agricultural Bioresources, South China Agricultural University, Guangzhou 510642, China*

^2^ *Institute of Quality Standard and Monitoring Technology for Agro-Products of Guangdong Academy of Agricultural Sciences, Guangzhou, 510640, China*

^3^ *Department of Botany, Division of Science and Technology, University of Education, Lahore 54770 Punjab, Pakistan*

^4^ *Guangxi Key Laboratory of Agricultural Resources Chemistry and Biotechnology, Yulin Normal University, 537000, China*

^5^ *Guangzhou Golden Rice Agricultural Science & Technology Co., Ltd., Guangzhou 510900, China*

^#^ Corresponding author: tangxr@scau.edu.cn; State Key Laboratory for Conservation and Utilization of Subtropical Agricultural Bioresources, South China Agricultural University, Guangzhou 510642, China


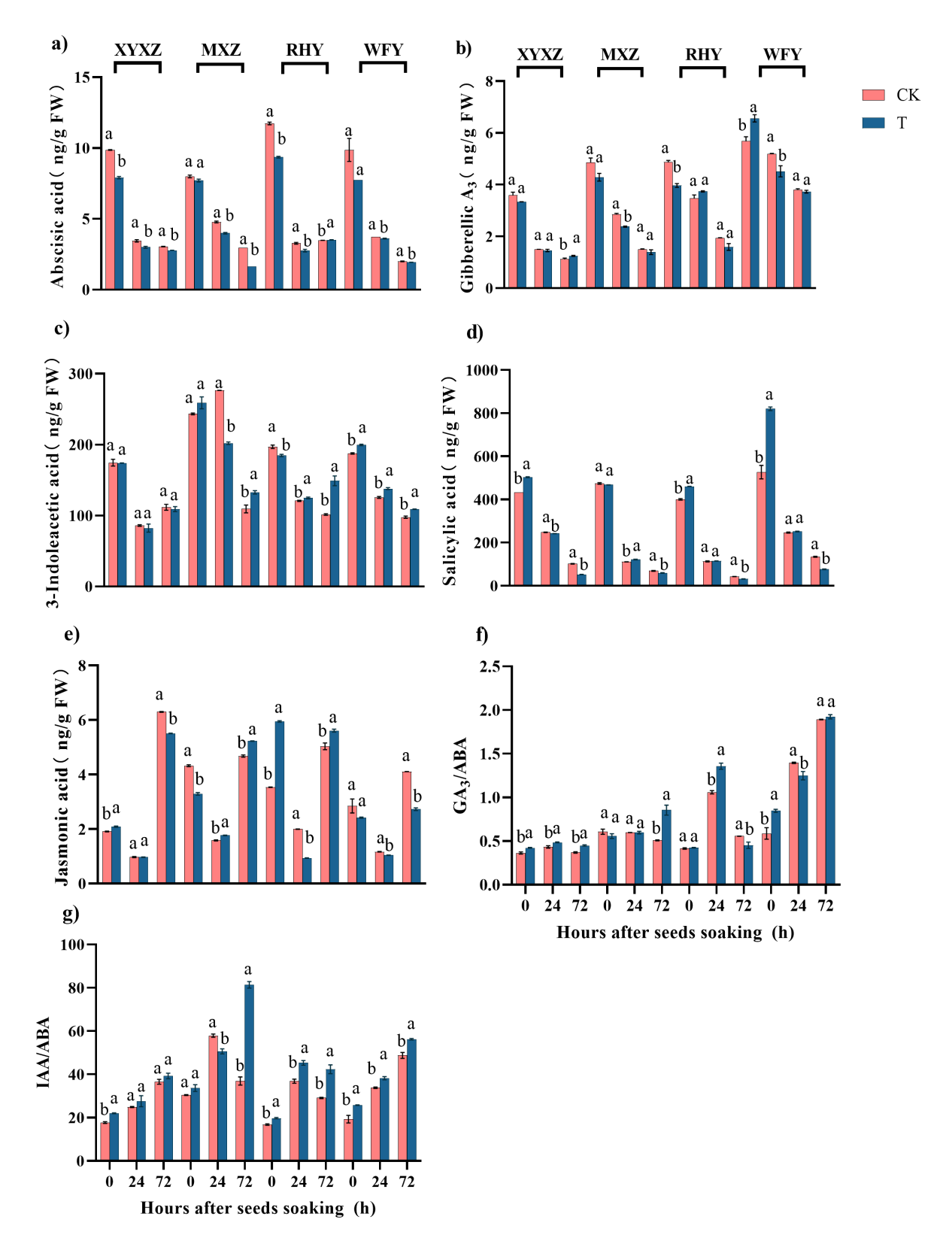


Figure S1. Effects of ultrasonic seed treatment on endogenous a) abscisic acid, b) gibberellic A_3_, c) 3-indoleacetic acid, d) salicylic acid, e) jasmonie acid content, f) GA_3_/ABA and g) IAA/ABA in seeds of four rice cultivars.

Each column represented the mean ± standard error (n=3). Bars sharing a common letter did not differ significantly at *p*<0.05. GA_3_/ABA: the ratio of gibberellic A_3_ and abscisic acid content, IAA/ABA: the ratio of 3-indoleacetic acid and abscisic acid content. CK: control treatment, T: ultrasonic seed treatment. XYXZ: Xiangyaxiangzhan, MXZ: Meixiangzhan 2, RHY: Ruanhuayou 6100, WFY: Wufengyou 615.


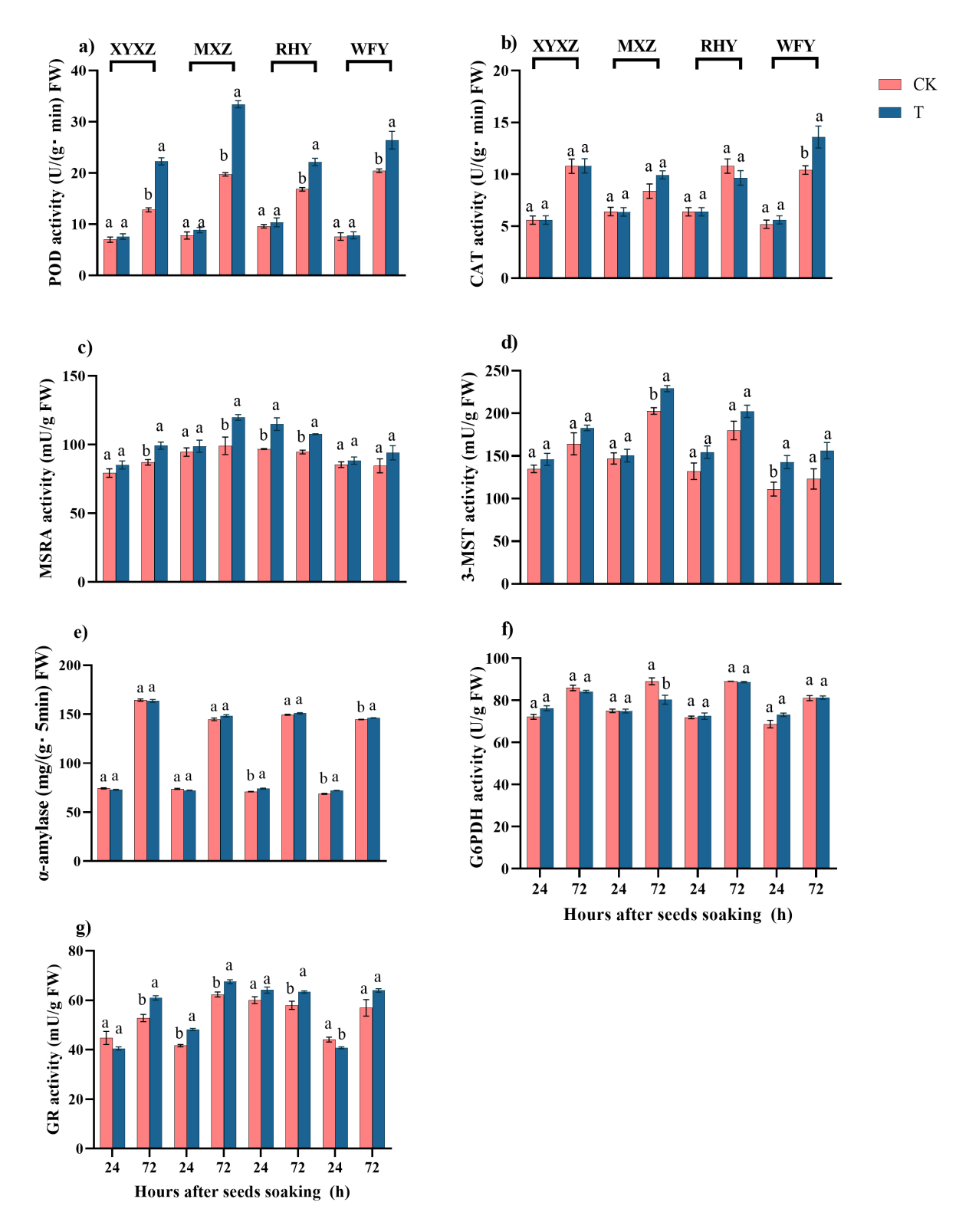


Figure S2. Effects of ultrasonic seed treatment on anti-oxidant enzyme activities of a) POD, b) CAT, c) MSRA, d) 3-MST, metabolic enzyme activities of e) α-amylase, f) G6PDH and g) GR in seeds of four cultivars.

Each column represented the mean ± standard error (n=3). Bars sharing a common letter did not differ significantly at *p*<0.05. POD: peroxidase, CAT: catalase, MSRA: methionine sulfoxide reductase, 3-MST: 3-mercaptopyruvate sulfurtransferase, G6PDH: glucose 6-phosphate dehydrogenase, GR: glutathione reductase. CK: control treatment, T: ultrasonic seed treatment. XYXZ: Xiangyaxiangzhan, MXZ: Meixiangzhan 2, RHY: Ruanhuayou 6100, WFY: Wufengyou 615.


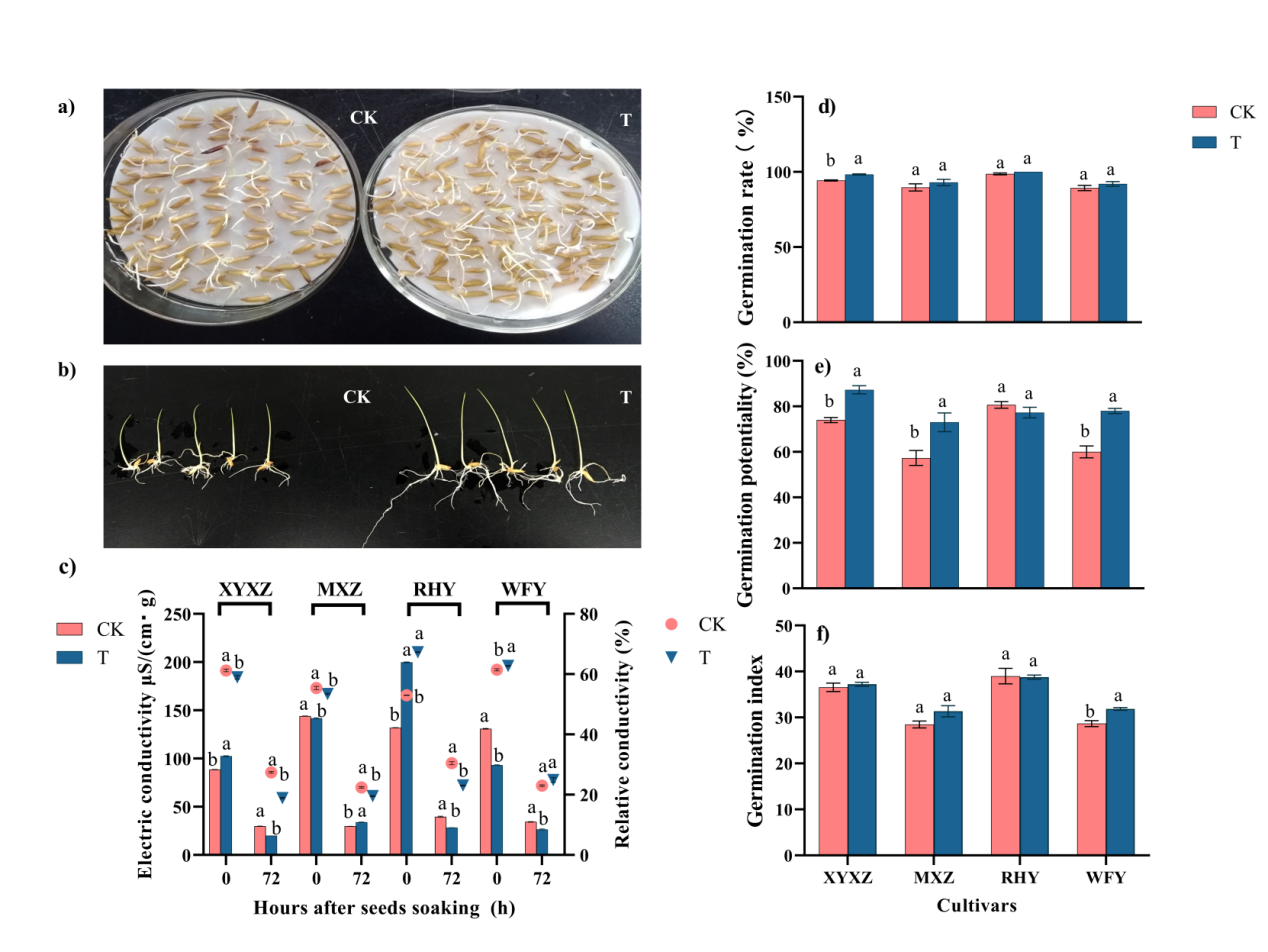


Figure S3. Effects of ultrasonic seed treatment on a), b) germination at 72 h and 120 h, c) electric conductivity, relative conductivity, d) germination rate, e) germination potentiality, and f) germination index.

Each column represented the mean ± standard error (n=3). Bars sharing a common letter did not differ significantly at *p*<0.05. CK: control treatment, T: ultrasonic seed treatment. XYXZ: Xiangyaxiangzhan, MXZ: Meixiangzhan 2, RHY: Ruanhuayou 6100, WFY: Wufengyou 615.


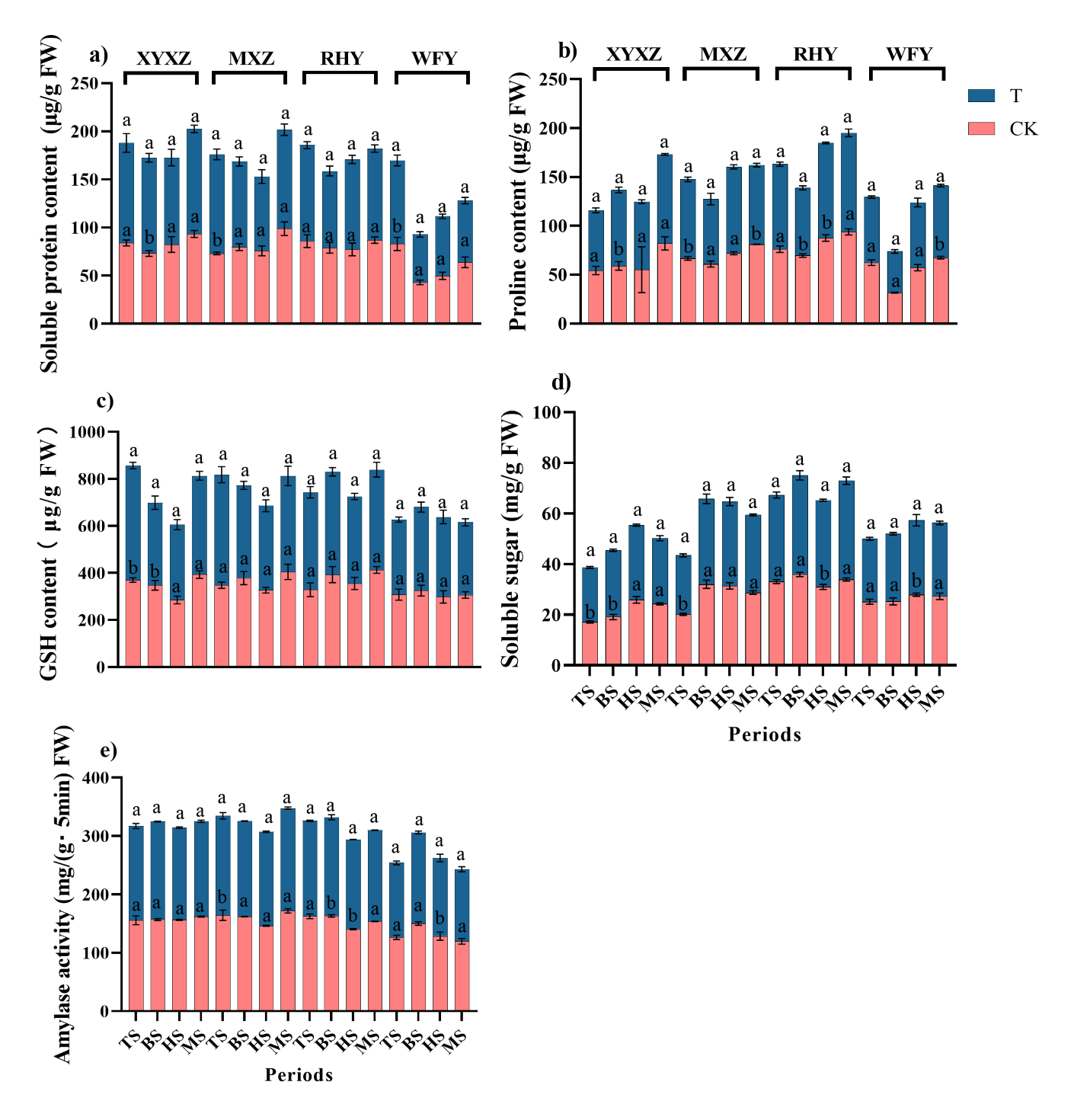


Figure S4. Effects of ultrasonic seed treatment on a) soluble protein, b) proline, c) GSH, d) soluble sugar content and e) amylase activity in flag leaf of four cultivars.

Each column represented the mean ± standard error (n=3). Bars sharing a common letter did not differ significantly at *p*<0.05. GSH: glutathione. CK: control treatment, T: ultrasonic seed treatment. XYXZ: Xiangyaxiangzhan, MXZ: Meixiangzhan 2, RHY: Ruanhuayou 6100, WFY: Wufengyou 615. TS: tillering stage, BS: booting stage, HS: heading stage, MS: maturity.


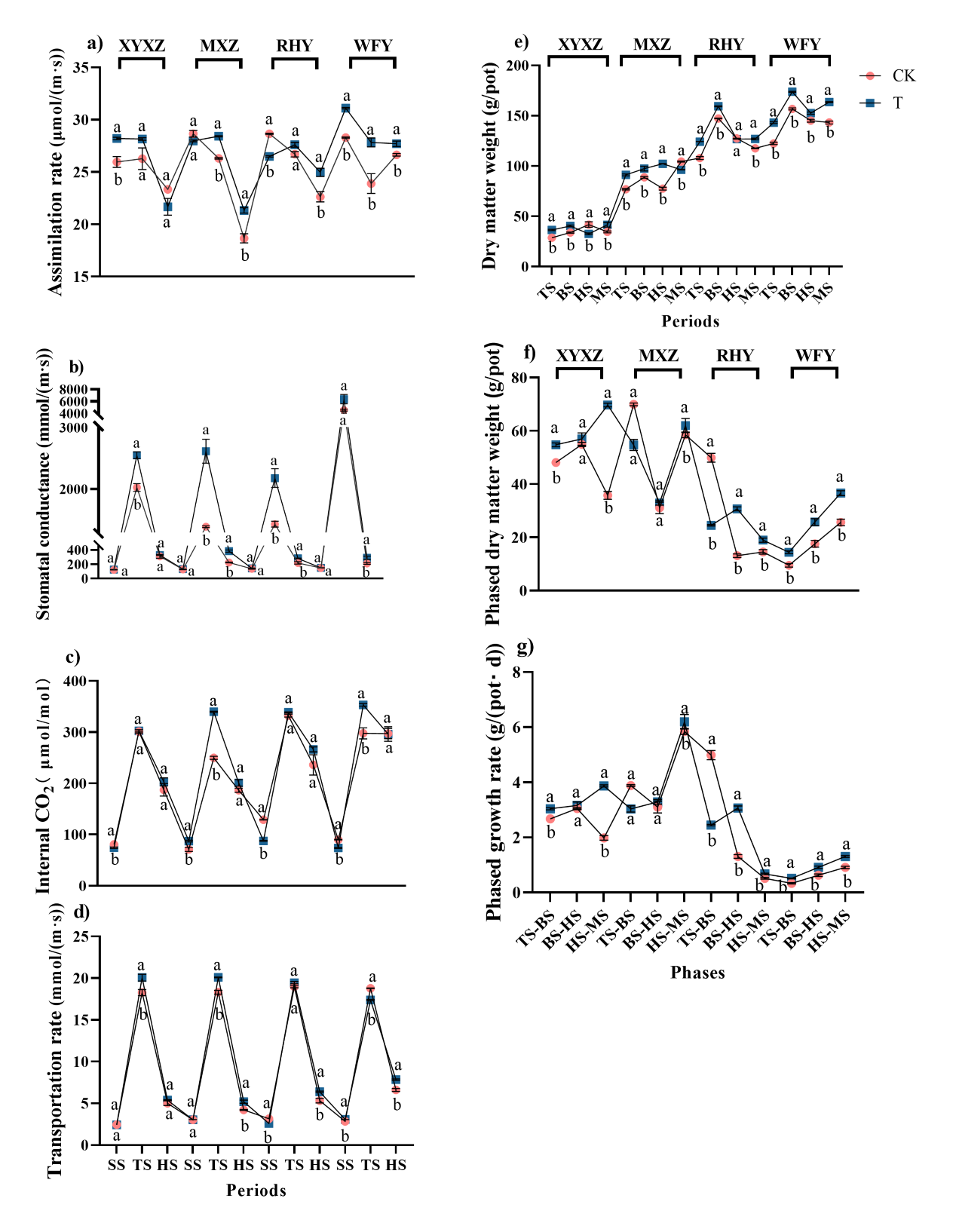


Figure S5. Effects of ultrasonic seed treatment on a) assimilation rate, b) stomatal conductance, c) internal CO_2_, d) transportation rate in flag leaf, e) dry matter weight, f) phased dry matter weight and g) phased growth rate of four cultivars.

Each column represented the mean ± standard error (n=3). Bars sharing a common letter did not differ significantly at *p*<0.05. CK: control treatment, T: ultrasonic seed treatment. XYXZ: Xiangyaxiangzhan, MXZ: Meixiangzhan 2, RHY: Ruanhuayou 6100, WFY: Wufengyou 615. TS: tillering stage, BS: booting stage, HS: heading stage, MS: maturity.


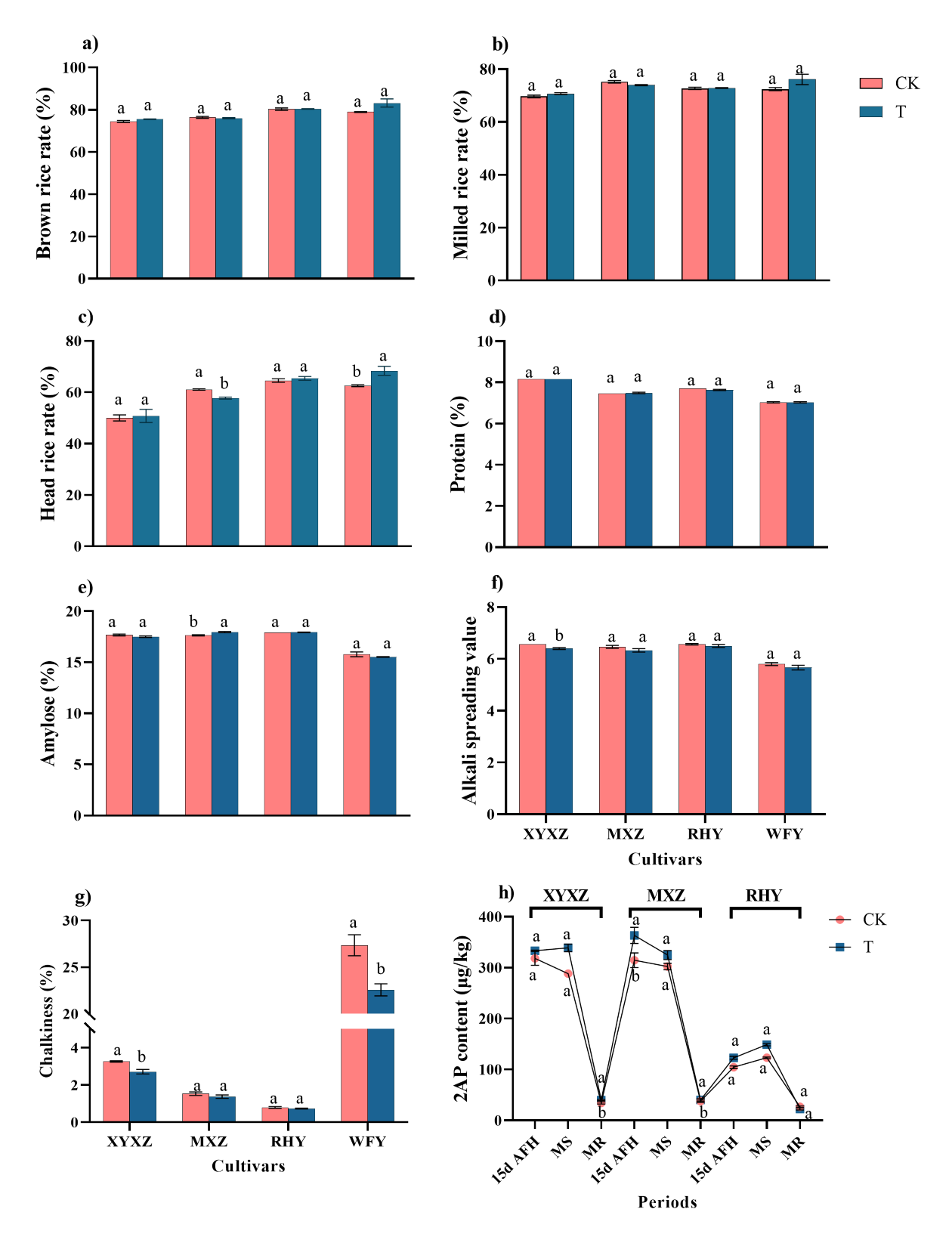


Figure S6. Effects of ultrasonic seed treatment on a) brown rice rate, b) milled rice rate, c) head rice rate, d) protein, e) amylose, f) alkali spreading value, g) chalkiness at maturity and h) 2AP content in grains at 15 d after full heading stage (15 d AFH), maturity (MS) and in milled rice (MR) of four cultivars.

Each column represented the mean ± standard error (n=3). Bars sharing a common letter did not differ significantly at *p*<0.05. CK: control treatment, T: ultrasonic seed treatment. XYXZ: Xiangyaxiangzhan, MXZ: Meixiangzhan 2, RHY: Ruanhuayou 6100, WFY: Wufengyou 615.


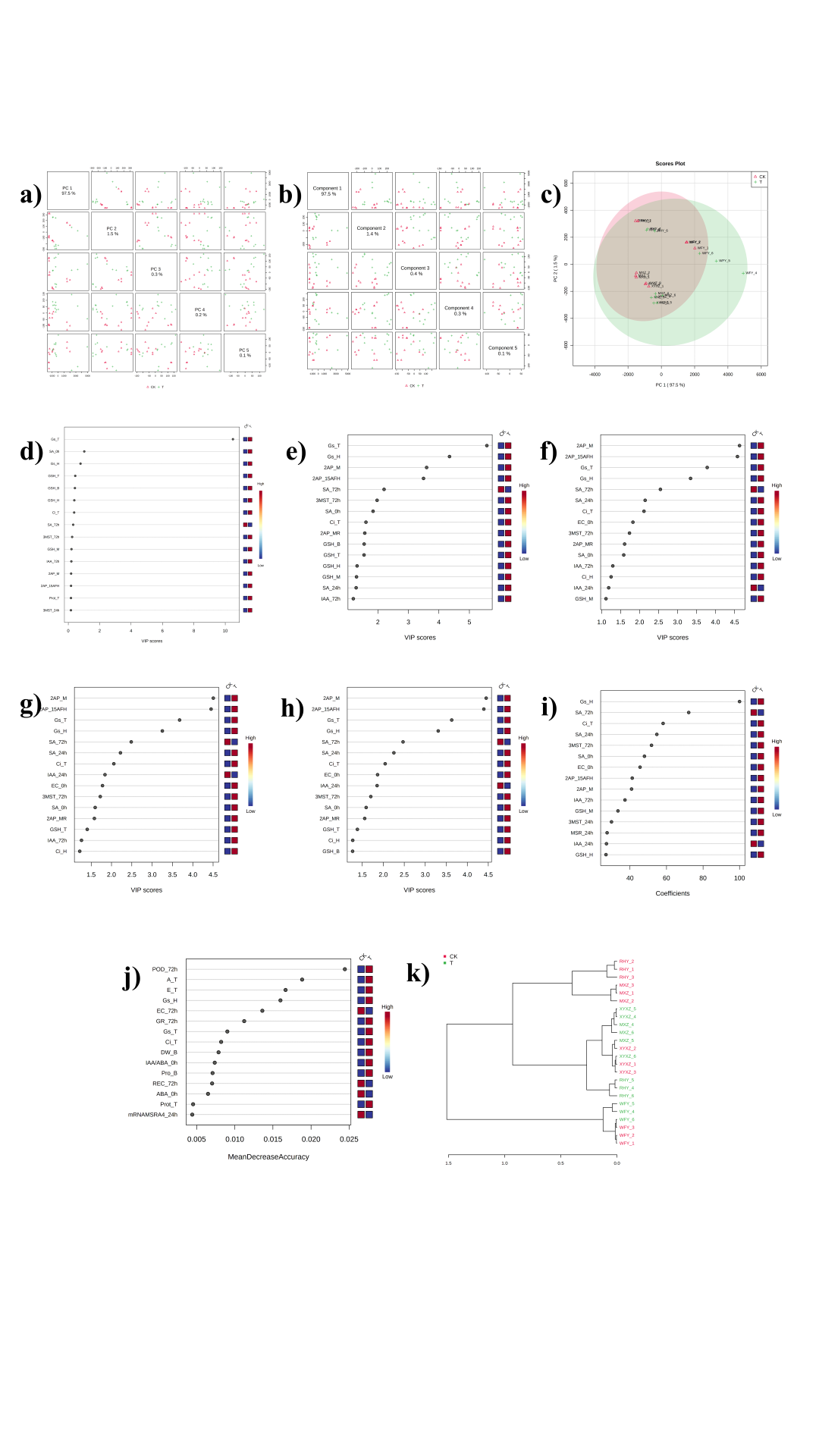


Figure S7. PCA analysis of the investigated parameters with a) PCA, b and c) PLS-DA, d-i) the important features to the 5 components analysis of PLS-DA for ultrasonic seed treatments, j) the parameters ranked by their contributions to classification accuracy (Mean Decrease Accuracy) and k) Hierarchical Clustering Dendrogram for rice cultivars.

CK: control treatment, T: ultrasonic seed treatment. XYXZ: Xiangyaxiangzhan, MXZ: Meixiangzhan 2, RHY: Ruanhuayou 6100, WFY: Wufengyou 615. EC_0h: electric conductivity in seeds at 0 h after seeds soaking, EC_72h: electric conductivity in seeds at 72 h after seeds soaking, REC_72h: relative conductivity in seeds at 72 h after seeds soaking, ABA_0h: abscisic acid content in seeds at 0 h after seeds soaking, IAA_24h: 3-indoleacetic acid content in seeds at 24 h after seeds soaking, IAA_72h: 3-indoleacetic acid content in seeds at 72 h after seeds soaking, IAA/ABA_0h: the ratio of 3-indoleacetic acid and abscisic acid content in seeds at 0 h after seeds soaking, SA_0h: salicylic acid content in seeds at 0 h after seeds soaking, SA_24h: salicylic acid content in seeds at 24 h after seeds soaking, SA_72h: salicylic acid content in seeds at 72 h after seeds soaking, mRNAMSRA4_24h: relative gene expression of *MSRA4* in seeds at 24 h after seeds soaking, MSR_24h: methionine sulfoxide reductase activity in seeds at 24 h after seeds soaking, 3MST_24h: 3-mercaptopyruvate sulfurtransferase activity in seeds at 24 h after seeds soaking, 3MST_72h: 3-mercaptopyruvate sulfurtransferase activity in seeds at 72 h after seeds soaking, POD_72h: peroxidase activity in seeds at 72 h after seeds soaking, GR_72h: glutathione reductase activity in seeds at 72 h after seeds soaking, GSH_T: glutathione content in flag leaf at tillering stage, GSH_B: glutathione content in flag leaf at booting stage, GSH_H: glutathione content in flag leaf at heading stage, GSH_M: glutathione content in flag leaf at maturity, Prot_T: soluble protein content in flag leaf at tillering stage, Pro_B: proline content in flag leaf at booting stage, A_T: assimilation rate in flag leaf at tillering stage, E_T: transportation rate in flag leaf at tillering stage, Gs_T: stomatal conductance in flag leaf at tillering stage, Gs_H: stomatal conductance in flag leaf at heading stage, Ci_T: internal CO_2_ in flag leaf at tillering stage, Ci_H: internal CO_2_ in flag leaf at heading stage, DW_B: dry matter weight at booting stage, 2AP_15AFH: 2AP content in grains at 15 d after full heading stage, 2AP_M: 2AP content in grains at maturity, 2AP_MR: 2AP content in milled rice.

| Table S1 Primer sequences of genes encoding enzymes involved in DNA and protein repairing | | | | | |
| --- | --- | --- | --- | --- | --- |
| Gene name | |  |  | Primer sequences | |
| 8-oxoguanine DNA glycosylase (OGG1) |  |  |  | F | 5'-CTCGATCAGAACCATGCTAT-3' |
|  |  |  |  | R | 5'-GTTTTGGGGTTAAGCTCTTG-3' |
| Protein L-isoaspartyl methyltransferase (PIMT1) | | | | F | 5'-ACCGCAGATTCCACAGGC-3' |
|  |  |  |  | R | 5'-GGGGCACATAGCGCACA-3' |
| Methionine sulfoxide reductase 2.1 (MSRA2.1) |  |  |  | F | 5'-GGTCGGGCATCTACTACTAC-3' |
|  | | |  | R | 5'-ATCTCCGTCACGATCCTCT-3' |
| Methionine sulfoxide reductase 4 (MSRA4) |  |  |  | F | 5'-TATCTACTACTACACCCCTGAGCA-3' |
|  |  |  |  | R | 5'-CCTGAACCCGAAGCGAC-3' |
| *Actin* |  |  |  | F | 5'-CTTCATAGGAATGGAAGCTGCGGGTA-3' |
|  |  |  |  | R | 5'-CGACCACCTTGATCTTCATGCTGCTA-3' |
